# Anti-microbial Use in Animals: How to Assess the Trade-offs

**DOI:** 10.1111/zph.12193

**Published:** 2015-04-22

**Authors:** J Rushton

**Affiliations:** Veterinary Epidemiology Economics and Public Health Group, Production and Population Health Department, Royal Veterinary CollegeHatfield, UK

**Keywords:** Antibiotics, economics, trade-offs, public and private goods

## Abstract

Antimicrobials are widely used in preventive and curative medicine in animals. Benefits from curative use are clear – it allows sick animals to be healthy with a gain in human welfare. The case for preventive use of antimicrobials is less clear cut with debates on the value of antimicrobials as growth promoters in the intensive livestock industries. The possible benefits from the use of antimicrobials need to be balanced against their cost and the increased risk of emergence of resistance due to their use in animals. The study examines the importance of animals in society and how the role and management of animals is changing including the use of antimicrobials. It proposes an economic framework to assess the trade-offs of anti-microbial use and examines the current level of data collection and analysis of these trade-offs. An exploratory review identifies a number of weaknesses. Rarely are we consistent in the frameworks applied to the economic assessment anti-microbial use in animals, which may well be due to gaps in data or the prejudices of the analysts. There is a need for more careful data collection that would allow information on (i) which species and production systems antimicrobials are used in, (ii) what active substance of antimicrobials and the application method and (iii) what dosage rates. The species need to include companion animals as well as the farmed animals as it is still not known how important direct versus indirect spread of resistance to humans is. In addition, research is needed on pricing antimicrobials used in animals to ensure that prices reflect production and marketing costs, the fixed costs of anti-microbial development and the externalities of resistance emergence. Overall, much work is needed to provide greater guidance to policy, and such work should be informed by rigorous data collection and analysis systems.

**Impacts**

- Benefits from antimicrobial use in animals should be compared against both their financial cost and the risks of antimicrobial resistance emergence
- Current data collection systems are inadequate to examine these costs and benefits
- Policy making on antimicrobial use in animals is therefore hindered

## Introduction

The use of antimicrobials in livestock production provides a basis for improving animal health and productivity. This in turn contributes to food security, food safety, animal welfare, protection of livelihoods and animal resources. However, there is increasing concern about levels of anti-microbial resistance in bacteria isolated from human, animal, food and environmental samples and how this relates to use of antimicrobials in livestock production. A common reaction is to assume that antimicrobials should not be used in livestock. This study will explore why there is a need for a balance between the contributions of the anti-microbial use to food security and safety with the costs of the increased risks of anti-microbial resistance emergence. Such trade-offs are not simple as there are different types of antimicrobials different uses of these drugs in livestock and food systems and a variability of management of animals.

The study looks first at the importance of animals in societies across the world, the changing management of these animals and the use of antimicrobials in the production systems – the context. It will then examine the economic frameworks available to assess the use of anti-microbials and how these could be applied – the theory. The following section provides an exploratory review of the data on the use of antimicrobials and the benefits and costs – the current evidence. Finally, there are some reflections on the gaps and why these gaps exist. The discussion focuses on the need for state interventions that balance the private short-term benefits against the medium- to long-term needs of society.

## The context – animals in society

**Fig 1:**
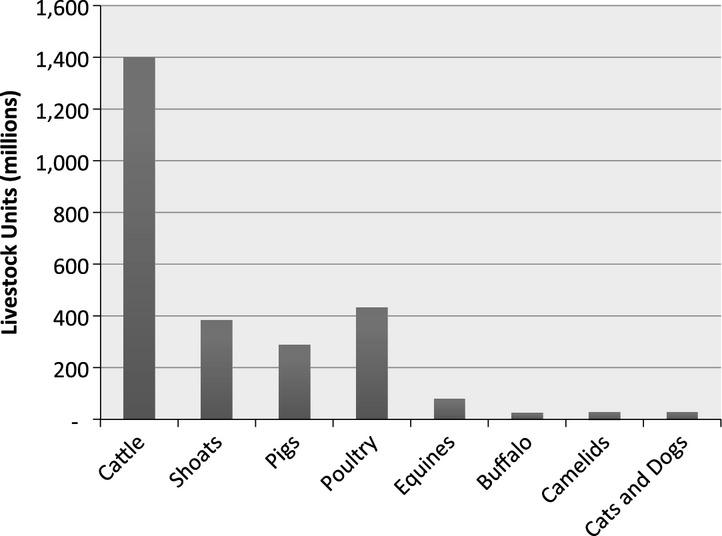
Global livestock units by species (FAO, 2014 data author analysis)

Animals are a fundamental aspect of societies around the world, and they feed people, provide pleasure and company, act as a store of wealth and, in many places, provide power to till land and to transport goods and people. In general, ‘human lives are enhanced by the use of animals’ (Norwood and Lusk, 2011). The sheer number of animals that humans have domesticated, with 7 billion people having 2.65 billion livestock units, underscores this importance.^1^ A majority of these domesticated animals are cattle, sheep, goats, pigs and poultry – livestock that are kept for food production, transport and draught power and as a form of investment (see Fig.1)^2^.

Across the world, there are approximately 0.38 livestock units, or an estimated 190 kilos of live animals per person. In species terms, this translates to three chickens, a third of a sheep or goat, a fifth of a cow, a seventh of a pig and a tenth of a cat or dog per person. The sheer scale of this biomass and the fact that these animals both compete for resources and share pathogenic and non-pathogenic organisms with humans demand attention to their role in relation to human health.

The role of animals in society is also changing and will continue to change in the future. Global human population is predicted to increase from 7 billion people in 2013 to 9.6 billion in 2050, and this population will be increasingly urbanised (Gerland et al., 2014). In general, urban populations are richer than rural ones, and richer populations demand greater amounts of meat relative to other food products. This creates demands on global food systems and more specifically livestock food systems. The response to the greater demand for livestock products has been a general increase in the global livestock populations. In addition, there is an intensification of livestock production systems with a reliance on diets of concentrated feeds, indoor housing and use of specialist breeds. These livestock populations are kept in higher densities clustered in areas with access to transport and processing systems. The animals are also sedentary rarely being allowed to scavenge or graze for food, rather food is brought to them. The final major change is that the livestock production systems are part of increasingly complex and lengthy livestock food systems with inputs such as grains coming from other countries and the livestock products being distributed across the world.

The changes in the way livestock are raised and processed have produced a dramatic rise in the availability of animal source foods (Steinfeld et al., 2006). This has undoubtedly contributed to an improved sense of well-being around the world through the greater access to reasonably priced livestock protein, employment with 1.3 billion people estimated to be employed in different livestock product value chains (Herrero et al., 2009), and the sheer number of households in developing countries involved in livestock earning activities. It has been estimated that approximately two-thirds of households in the developing countries earn income from livestock (Davis et al., 2010).

Therefore, animals are important in societies across the world and livestock, in particular, are critical to food systems. Animals are involved in everything we do; they compete for resources such as land and water, and they pose risks because the diseases they contract can be transferred to humans. The role of animals in society has changed and continues to do so. There is a tendency towards a greater intensity with which livestock are kept, many being largely sedentary and reliant on concentrated feeds. These changes have given rise to different animal health problems and the need for different systems of health management. Part of these changes in management relate to the use of antibiotics in livestock production, which includes the use of antibiotics as either therapeutic, metaphylactic or prophylactic measure. Increasingly, questions are being raised as to whether the use of antibiotics is justified, and some countries have modified their legislation in response to problems of anti-microbial residues and emergence of anti-microbial resistance. Thus, policies to manage animals and the diseases they suffer from remain critical.

The following sections will provide a suggested framework to examine the trade-offs in the use of antimicrobials in livestock production. The intention is to provide some clarity in this debate and also stimulate discussion around a very difficult policy area of anti-microbial use.

## Proposed economic frameworks to assess anti-microbial use in animals

Anti-microbial use in livestock production is accepted and promoted to achieve livestock production and animal welfare goals at both an individual and societal level. The benefits from the sale of antimicrobials accrue to the pharmaceutical companies and the animal health professionals involved in their distribution. The livestock producers benefit from their ability to raise and harvest livestock products with more certainty and high levels of productivity. Beyond the farm gate the slaughter and food processing industries have benefits relating to more uniform animals that are supplied in numbers that have low variation. Finally, the consumers enjoy access to livestock products that are of high quality, in quantities and price that make them accessible.

**Fig 2:**
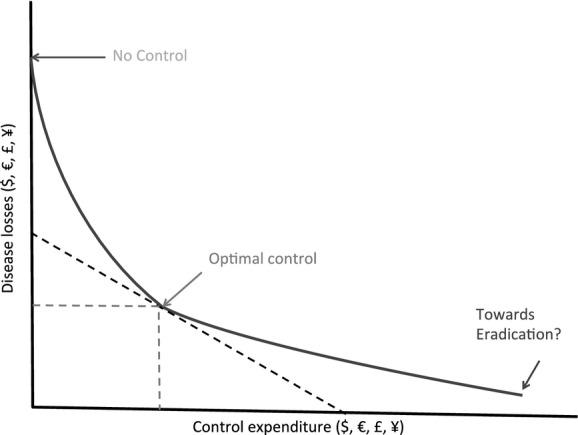
Disease Loss – Expenditure Frontier (adapted from Mclnerney, 1996)

How do we balance the benefits from antimicrobials with the possible negative implications that could occur with misuse and overuse of antimicrobials. Economists have explored these trade-offs in animal health over the last 40 years. Mclnerney (1996) applied a theoretical production economics framework to animal disease comparing losses in production with expenditure on control. The greater the losses the lower the expenditure with a relationship between the two. Such a framework is useful in considering how a farmer would apply antimicrobials in their production system, the antimicrobials are an expenditure aimed at reducing losses in production. The level of application will not necessarily lead to the complete removal of the animal health problem, rather there is a point of equilibrium that relates to the value of the losses avoided and the costs of the treatment. Figure2 indicates some of the main points underpinning such a relationship.

**Fig 3:**
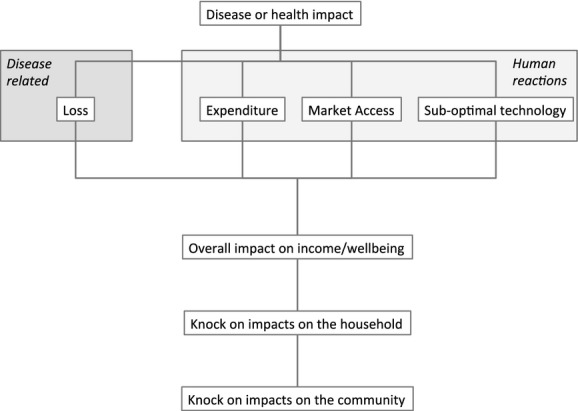
Proposed economic framework to assess an animal health problem such as the use of antimicrobials in animals.

In a less theoretical framework, Rushton et al. (1999) disaggregate animal disease impacts. They identified direct and indirect impacts – the former relating to McInerney's disease losses and the latter being control expenditures related to human reaction to disease presence and risk. A further modification is suggested in this study by separating human reactions to animal health problems into three categories:

- purchasing of medicines (including antimicrobials), vaccines, services and diagnostics – expenditure;
- closing or restricting of markets due to the presence of anti-microbial residues – market access;
- the use of suboptimal technologies which may be the case if anti-microbial leads to resistance and the need to have breeds and management systems that have a lower overall productivity – suboptimal technology;

Human reactions to problems related to anti-microbial use have an impact on disease losses, and there is a balancing point required where sufficient antimicrobials are used to optimise production goals for society. Figure3 presents a possible framework for this structure with an overall impact on society.

When developing a framework, one must recognise that not all expenditure costs are equal. Some relate directly to an animal disease management process and could be defined as variable costs, which are defined as costs that vary according to the level of production and are specific to a livestock enterprise (Rushton, 2009). Anti-microbial use at farm level would be a variable cost. Others cannot be so easily assigned and relate to the development of infrastructure, training and organisational capacity in general, which are defined as fixed costs that cannot easily be assigned to an activity and are investments that are made to last for extended period of time (Rushton, 2009). For example, the systems required to monitor both anti-microbial residues and anti-microbial resistance require the development of sampling procedures, protocols for sample storage and laboratories that can carry out tests. There is also of course a major cost in the development and testing of new antimicrobials, and as evidenced by the lack of recent new antimicrobials, these costs have become very large (Davies, 2013).

**Fig 4:**
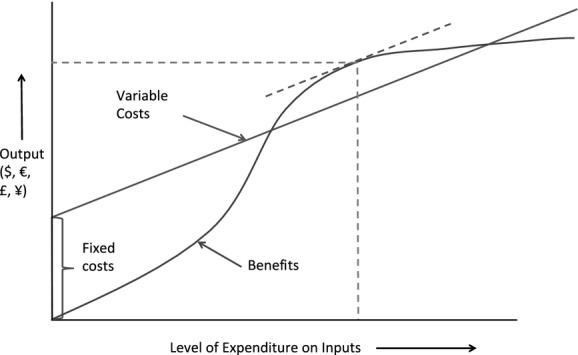
Cost-benefit model for livestock disease control with fixed costs (adapted fromTisdell, 2009)

These larger fixed costs are borne across society and require a modification of the frameworks proposed by McInerney that largely focuses on smaller, variable costs. Tisdell (2009) proposed that countries that do not invest in fixed cost elements of their animal health systems would find it difficult to incorporate and succeed with individual disease management campaigns. The development of antimicrobials and the process to monitor anti-microbial use, residues in animals, food and the environment and the changes in anti-microbial resistance requires significant societal investment that is beyond the capacity of many countries. Tisdell (2009) developed a theoretical framework around his arguments (see Fig.4).

The value of these impact assessments is that they identify weaknesses in the management of antimicrobials and the potential impacts in terms of residues or resistance emergence. A policy change (*e.g*. legislation and/or direct interventions) requires tools that examine such a change, and the preferred tool for such assessments is a cost-benefit analysis. This examines marginal or additional changes in costs and benefits over time and assesses the economic profitability of a given change.

**Fig 5:**
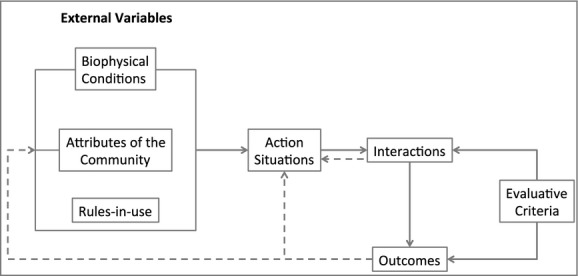
Institutional analysis and development framework (modified from Ostrom, 2010)

Theories based on production economics focus on rationality and optimal solutions. A change over time requires a time value for money in different periods which involves a value judgement that can either be individual or societal. The latter is often a decision made by policy makers and can differ according to whether an investment is for a productive or consumption activity. In addition, lengthening the horizon of a changes leads to uncertainty in the outcomes of the change, and this creates additional problems of fitting the theory to reality. One final complication is the institutional environment^3^ in which a change takes place that can affect how differ people involved in the change can value resources used. Overall, this added complexity requires that backbone of theory needs to be softened, it needs to look at the wider institutional environment in which decisions are taken (Hennessy, 2013). A suggested way forward would be to place anti-microbial use in an institutional analysis and development (IAD) framework (see Fig.5).

The IAD framework has been successfully applied to investigate common good management such as water, grazing and security. It focuses not simply on the economic incentives that focus on the balance between the losses caused by the health problem and the expenditure and other reactions of people to the presence of that problem. It adds to the analysis to question how rules in society affect how resources and people's time are valued and how this can affect decision-making within different components of society to modify people's behaviour. This framework captures not only the additional costs and benefits of managing a health problem but also questions the very way society can modify how important resources in society are valued. An example would be the use of taxation policy for antimicrobials affecting the price farmers pay in their livestock operations. The framework therefore would seem to be a useful way to examine antimicrobials which could be considered a common good that can be affected by misuse and/or overuse.

In a more complex example, if anti-microbial resistance was clearly shown to pass through the food system, then a challenge is where to intervene within that system. An important aspect will be to assess the technical feasibility of interventions and then to look at whether these measures will be considered cost-effective and socially beneficial. In complex food systems, questions need to be raised on:

- Can implementation of the intervention be verified within the food system by people affected? that is Can moral hazard be reduced? (Wolf, 2013)
- How will people's decision-making be affected by the intervention? that is What do we understand of human behaviour?
- What do we know of the rule breakers? which requires an understanding of the institutional setting in which rule breaking is encouraged, not just that some people are rule breakers.

In an example of the difficulties of looking at the biology in the livestock food systems that are now common across the world, there appears to be a growing recognition that resistance emergence can have three possible routes. The first is an underlying process of resistance that has always been in place (D'Costa et al., 2011). Secondly, in an environment where livestock are raised with the use of antimicrobials there will be evolutionary pressure for bacteria to carry and express resistance genes; if the antimicrobials are withdrawn from the production systems, this resistance will reduce. A third emerging area of concern is through the transmission across different groups of animals. This is particularly important in the types of breeding structures that are used in poultry systems where there are pureline, grandparent and parent flocks and production birds. These birds are kept in separate units, and are under different management systems and in many cases under different ownership. What appears to be a healthy bird entering a system has been shown to carry and transmit resistance such as extended-spectrum beta-lactamase-producing bacteria (Dierikx et al., 2013). Given that these populations are shaped in a pyramid, there is evidence that changes in the top of the pyramid can influence the bacteria with resistance genes and phenotypes in the much more numerous production birds (Laube et al., 2013). This example demonstrates that actions of people in the complex food chain can influence the resistance burdens on others animals and also create risks to humans working with animals (Huijbers et al., 2014) and across the food system (Egervärn et al., 2014). These issues can be both dramatic in terms of scale and have geographical implications for the spread as many countries import birds from overseas breeding flocks.

The food system therefore needs to be pulled apart in terms of who is involved in manufacturing, distributing, supplying and using antimicrobials. Once in an animal the likelihood of anti-microbial residue and resistance with a system in place to capture these changes. These technical outcomes need to be translated into probabilities of affecting the health of animals and humans through the lower usefulness of the antimicrobials available.

## Data on the economic assessment of antimicrobials in animals

### The benefits

In pig and poultry systems, and in countries where this practice is allowed, it is common that antimicrobials are used as growth promoters (Dibner and Richards, 2005; Castanon, 2007). The potential growth promoter effect of antimicrobials was discovered in the 1940s, when it was observed that when healthy animals were fed dried mycelia of *Streptomyces aureofaciens* containing chlortetracycline residues, their growth improved. The same approach was advocated in the mid-1950s, as researchers found that small, subtherapeutic quantities of antimicrobials used as feed additives decreased the time and total feed needed to grow an animal to market weight (Marshall and Levy, 2011).

The exact mechanism by which the antimicrobials promote greater efficiency of feed use and hence growth has never been fully clarified (Pagel and Gautier, 2012), reflecting the complexity of the impact of antimicrobials on the microbiome and its interaction with the animal's physiological body functions. As the level of gut absorption of some of the antimicrobials used as growth promoters is reduced (Dibner and Richards, 2005), the actual mechanism of action must be at the gut level (Dibner and Richards, 2005). These can include direct effect on the microflora leading to decreased competition for nutrients, reduction in microbial metabolites that depress growth and a reduction in opportunistic pathogens and subclinical infections (Dibner and Richards, 2005). Some of the more recent theories point to a non-anti-microbial but anti-inflammatory effect in the gut (Niewold, 2007), modulation of gut immune responses (Costa et al., 2011) or subtle changes in population composition of the gut microbiome (Danzeisen et al., 2011).

Data on the faster growth generated by increasing consumption of antimicrobials for growth promotion have been published and provide a convincing argument for use in pigs and poultry, particularly during the early stages of life (Thomke and Elwinger, 1993) and under poor hygiene conditions (SOU, 1997). The differences in growth rates between animals consuming and not consuming AGP have been more difficult to identify in production systems where hygiene conditions were changed with regard to improvements in housing, feed and water. There is increasing evidence of there being little value of AGP in livestock production systems that have improved hygiene standards, and the use of anti-microbial growth promoters (AGPs) in poultry units in the US actually reduces profit margins (Graham et al., 2007). As a note of caution to what a appears to be a growing consensus a recent study indicates that improvements can be found in layer birds (Liu et al., 2014), the context of the production system is not clear and the ability to compare studies requires more information.

The increasing awareness of the risk of resistance led to the ban of growth promotion use in Europe. Despite such bans, there are ways that production systems can receive antimicrobials at low levels, and there is a need to look more carefully at the economic incentives and the institutional environment. In addition, the actual data on the effect of the use of antimicrobials for growth promotion were published some time ago (SOU, 1997; Thomke and Science, 1993) and appear not to have been updated even though feed quality, management and housing have improved considerably. Therefore, the actual benefits of one of the major uses of antimicrobials in livestock production are unclear, and evidence compiled so far from Europe (Cogliani et al., 2011) and the US (Maron et al., 2013) indicates that anti-microbial use could be reduced with changes in management and with minimal impacts on livestock production levels. Whilst some argue that Europe no longer uses antimicrobials for growth promotion, there is evidence that many animals are treated in batches where only some animals are sick. The trigger for these treatments leads to metaphylactic use. Evidence of this problem can be seen in the need for the Dutch and Danish governments to change their legislation and enforcement procedures to reduce anti-microbial use in livestock production systems. This change in the institutional environment is an important point of reference for any successful management programme.

In summary, the benefits of the use of antimicrobials for curative medicine are much clearer – livestock that are sick can be made healthy and productive again. The case on the improved performance of healthy livestock with low levels of anti-microbial use is less clear cut.

### The costs

The possible benefits from the use of antimicrobials in animals need to be balanced against their cost and the costs of application and the costs in humans and animals caused by increased risk of emergence of resistance. The study will not review the monetary costs of antimicrobials and their application, albeit this is an important element, rather it will focus on the externality issues that relate to the increased risks of the emergence of resistance.

There are three potential routes that anti-microbial resistance could spread from livestock production systems: (i) through the food system, (ii) direct contact between people and animals and (iii) through environmental contamination. These are discussed in the following sections, and information is also provided on the potentials costs to human health if resistance is passed to humans.

#### Food system transmission and direct contact

As with most public health problems, the initial reaction to problems is to focus on the food system to ensure that consumers are not affected. The commission who have drawn up Codex Alimentarius have generated guidelines on a structured risk analysis framework that addresses the risks to human health associated with the presence in food of bacteria carrying resistance genes or resistant phenotypes that is linked to the use of antimicrobials in animals or food preparation. Within the limits of the need for compromises between members of the commission, these guidelines also provide advice on management strategies to reduce such risks (Codex Alimentarius, 2011).

The use of fluoroquinolones (e.g. enrofloxacin) in food animals has been linked to the development of ciprofloxacin-resistant *Salmonella*, *Campylobacter* and *E. coli*, which were responsible for human infections. Resistance generated to these antimicrobials through the use in animals is a part of the resistance profile as these antimicrobials are widely used in human medicine and the spread can be through travel and food contamination. However, several reports suggest that multiple *E. coli* human infections may have originated in food animals, mainly poultry (Johnson et al., 2007; Warren et al., 2008).

In terms of looking at the impacts on the human health McEwen (2012) published a review paper, summarising the available American quantitative human health risk assessments of anti-microbial use in animals (McEwen, 2012). Risk estimates ranged from a few additional illnesses per million at risk, to many thousands. Comparison between studies is however far from linear, as few of them consider the same drug/bacterium combination or the same risk question, and the methodologies used also differ substantially (McEwen, 2012). Similar to the issue on the value of antimicrobials for growth promotion, there is little emerging consensus.

In general, there are a number of studies that indicate an association between anti-microbial use in livestock and resistance in bacteria. However, few have quantified what this subsequently means in terms of public health, which suggest that those that fund the research do not have a holistic picture in terms of the impact of resistance emergence. Hence, there is a lack of data and information which can lead to uninformed policy making at international and national levels, poor development of private standards and uninformed choice of production systems at farm level. Snary et al. (2004) indicated that much data are available for food system risk assessments of resistance, but it is rarely in a format that allows strong quantitative analysis. The Codex Alimentarius guidelines on assessing anti-microbial resistance risks in the food system are important in this context as they provide an analytical structure to guide data collection and enhance data capture.

There is a gap in the data on the relative importance between the transmission of bacteria with resistance genes and phenotypes to humans from animals through direct contact versus through the food system. Understandably, this will tend to be context specific and dependent on the production system, the use of antimicrobials and the effectiveness of the food system to manage bacterial burdens. Yet overall the literature is poor in this critical area of managing anti-microbial resistance.

#### Environmental contamination

There are also environmental risks associated with anti-microbial usage: (i) the hazard of emission of antimicrobials into the environment, for example a significant quantity (75–90%) of tetracycline used in food animals is excreted largely unmetabolised into the environment and (Chee-Sanford et al., 2001) (ii) the hazard of bacteria with resistance genes being disseminated into the environment when manure and urine from livestock production are spread. The data on this dissemination are limited and require further work to draw hard conclusions. An additional concern is the waste water from pharmaceutical manufacturers which if left untreated has been shown to create pockets of resistance.^4^

In summary, most antimicrobials given to livestock are excreted. Their impact can be localised in terms of influencing the microbiome of the animal and also more generalised through the anti-microbial coming into contact with the environment as it is excreted in the manure and urine. Some data indicate the problems this appears to cause with an association between the spread of manure and the existence of resistance genes in the environment (Wegener, 2012). Again, there are gaps in our knowledge of the overall impact of the environmental externality created using antimicrobials in livestock systems. There are also gaps in understanding of the emergence of resistance from farm systems are largely localised in terms of direct contact with the animals or the environment they operate in rather than through food borne spread. This aspect is particularly critical when we consider that little if anything has been carried out with regard to the use of antimicrobials in companion animals which are a smaller overall number and biomass but are more frequently in direct contact with people.

#### Estimated costs of anti-microbial resistance in human

In terms of calculating the costs of anti-microbial resistance, there are studies that have attempted to estimate the monetary externalities of resistance. Kaier and Frank (2010) measured the externality of anti-bacterial use in human medicine and concluded that consumption of a single defined daily dose of second-generation cephalosporins, third-generation cephalosporins, fluoroquinolones and lincosamides is associated with a negative externality of about EUR 5, EUR 15, EUR 11 and EUR 12, respectively. This estimate relates to increased likelihood of the emergence of resistance, and the cost increases in health care and human health loss associated with that increased resistance (Kaier and Frank, 2010).

In contrast, use of one litre of alcohol-based hand rub solution for hand disinfection is associated with a positive externality of about EUR 61. Kaier and Moog (2012) concluded that a 32% reduction in the cost of MRSA to the German healthcare system could be reached, if the use of fluoroquinolones and third-generation cephalosporins (in humans) was reduced by 10%, together with the same increase in the use of antiseptics for hand disinfection (Kaier and Moog, 2012). Tansarli et al. (2013) looked at the in-hospital costs attributable to anti-microbial multidrug resistance on (human) inpatient care cost and concluded that these costs are alarmingly high (Tansarli et al., 2013). For example, with respect to MRSA, the attributable mean total costs per patient varied from USD 1014 to 40 090, and they varied from USD 1584 to 30 093 among studies on extended-spectrum β-lactamase-producing *Enterobacteriaceae*. The large spread on the estimates relates to uncertainties on the parameters and outcomes in individual cases.

### Overall economic analysis

From an economic perspective, it is important to recognise that low-level anti-microbial use in livestock influences the efficiency of feed inputs and hence the overall productivity of a system. Yet there are trade-offs in terms of animal health. For example, whilst antimicrobials may enhance the growth and efficiency of livestock, it could well lead over time to the emergence of resistance to antimicrobials and any outbreaks of disease of organisms with resistance genes would require the use of more expensive antimicrobials. Conversely not using anti-microbial prophylactically may increase feed costs and perhaps costs associated with disease and death loss, but diseases are less likely to be caused by resistant pathogens and can often be treated with less expensive first-line anti-microbial drugs (Mathews, 2001). The balance between the short-term gains from using antimicrobials prophylactically versus medium to long-term costs of resistance build up illustrate in a localised sense the trade-offs that need to be made at animal production level.

There are few studies that have attempted to look at the trade-offs between the costs of antimicrobials and the benefits gained. Collignon et al. (2005) estimated that the use of antimicrobials in livestock production does little for malnutrition yet there analysis focussed on the use for growth promoters rather more general use of antimicrobials that would include curative treatments. Graham et al. (2007) carried out a trial in a large poultry producer in the US where anti-microbial growth promoters were reduced. They found that the use of antimicrobials in this way created a cost to production, not a benefit. Vågsholm and Höjgård (2010) presented a careful analysis of how the externalities need to be incorporated into a taxation mechanism on anti-microbial pricing. Their analysis is useful in putting into context the need for government policies on taxation of antimicrobials and the fact that this element of policy making should be aiming to rectify market failure. Smith et al. (2006) highlighted that much work is done on resistance issues in terms of micro-level impacts and interventions to avoid or minimise the risks. They argued that macro-level impacts needed to be examined carefully for wider implications on the economy, as evaluations tended to concentrate on the economic impact to the healthcare sector alone, with poor estimation of the social costs and benefits of a disease or intervention. Further work indicated (Smith and Coast, 2013) that an increase in resistant organisms coupled with no new anti-bacterial discovered since 1987 (Davies, 2013), and very few antivirals and anti-fungals indicate a crisis. The outcome could be a need to change how current human and animal health systems manage infectious diseases in the future. Funding programmes are beginning to react to this challenge, and it remains to be seen how this will evolve. What is clear is that currently available estimates of the economic costs of anti-microbial resistance fail to recognise that antimicrobials are integral to modern health care.

Overall for an area of society that is so important – animals and antimicrobials there is paucity of data and information on changes in production, costs of use and externalities created by changes in resistance levels. This makes economic analysis difficult and when attempted the answers generated are based on many assumptions.

## Suggestions for the future

Impact assessment frameworks are needed to identify bottlenecks in animal health and welfare management. In addition, it is important that such impact assessments collect and document the public and private expenditure (Gilbert and Rushton, 2014) on animal health and disease management in order to generate information on usage of antimicrobials and for economic analysis to have cost profiles. Remembering that not all antimicrobials are the same (Acar et al., 2012) and will have varying social and economic impacts due to their different biological actions, different roles in animal and human medicine and resistance against a particularly group of antimicrobials. Many governments currently focus their work on public expenditure which is a partial and limited picture of expenditure across a society, and there is a general lack of data that allow fine detail of anti-microbial use to be specified. In order to achieve a more complete picture the impact assessment frameworks need to direct national and international data collection efforts which should include species and systems they are applied to the type of antibiotic and the dosing. The species needs to cover companion animals as well as food animals as it is still unknown how important they are in the general management of resistance.

**Fig 6:**
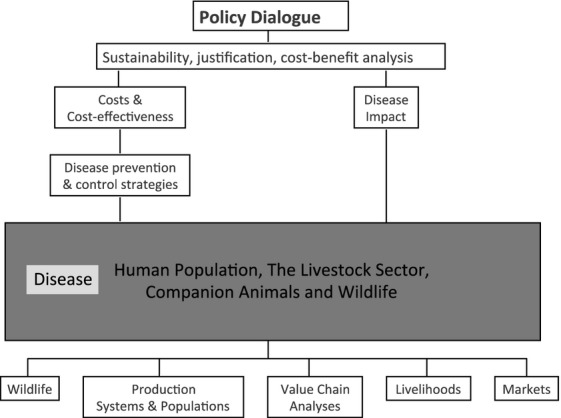
Elements required for building a sound economic assessment of One Health and welfare problems.

On the costs side, more attention is required on the large fixed costs required for the development of new antimicrobials and in the need for pricing mechanisms that reflect the need to cover these costs and manage resistance (Vågsholm and Höjgård, 2010). There must be clear information on the capacity of the private sector to manage fixed costs, and this is particularly relevant in situations where livestock sectors are becoming integrated with a small number of large companies. Economics need to be incorporated in epidemiological models, as well as in the monitoring and evaluation of animal health projects and programs. The state's role must be better defined with regard to coordination, legislation and investment in research and information provision. One must also understand that cost-benefit analysis only provides an estimate of economic profitability. Overall, good policy dialogue needs to build on data from different areas of the economy, as well as analysis that incorporates biological, technical and economic disciplines. Figure6 presents a summary of this approach applied to a One health and welfare perspective. This framework includes the use of antimicrobials and the emergence of resistance.

## Conclusion

Livestock health is important to societies across the world. Economic analysis of animal health is complex and disease dependent. This complexity only increases at a policy level and requires a systems approach with interdisciplinary working. Such analysis must account for the roles that animals play in society and the prices of resources they compete for. This implies that a realistic assessment of costs and benefits from animal health policy making will be complex. The communication of results should focus on what decisions need to be made and why, using economic principles to focus on resource allocation. Wider societal issues such as social acceptability and political palatability should also be considered and included. Once programmes are established, they must be regularly reviewed with the same rigour to avoid institutionalisation.

Livestock are an important component of societies across the world, yet their role is changing and the systems in which we keep livestock have become more intensive. Part of this change in production has been an increase use of antimicrobials in the management of animal health and in some cases to increase the efficiency of feed conversion in the animals. Antimicrobials have therefore become integral to the livestock systems that the world is increasingly dependent on. Yet the amount and frequency of use of antimicrobials across the world is different even in systems with similar levels of intensification. These differences in use are related to the rules and enforcement of anti-microbial use in livestock. This institutional environment is also evolving as anti-microbial resistance has become associated with the use of antimicrobials in livestock production. The emergence of resistance could be through the food systems, which are increasingly global in their reach, and can be through local contact and environmental contamination.

There is an awareness of these complexities in the use of antimicrobials the emergence or resistance and the difficulties of rigorous economic assessment. The study presents a framework that could assist in approaching this through the division of impacts into impacts caused by a health problem across species and the costs of people's various reactions to such a problem. It also raises the need to understand the institutional environment in which prices are set for key resources and services. Applying only part of this framework in an exploratory review of the literature on the costs and benefits of anti-microbial use in animals indicates literally the cupboard is bare. There are gaps on the impacts of low doses of antimicrobials used as growth promoters. The data on attribution of resistance emergence from the livestock production unit and through into the food system and the environment are limited. Plus the data on the economic impact of resistance in human health are variable. As stated earlier currently available estimates of the economic costs of anti-microbial resistance fail to recognise that antimicrobials are integral to modern health care.

Antimicrobials resistance represents a global societal problem, a good example of a ‘Tragedy of the Commons’: individuals are depleting a shared ‘global public good’ by acting short term out of self-interest, even if doing so runs contrary to the overall long-term best interest. This is generating a response from different organisations involved in safe guarding public health, yet there are major gaps in knowledge, data and information. These gaps create weaknesses in terms of decision-making on international and national public policy and also in setting private standards across livestock food systems. If the debate on what to do next to find solutions to this problem is to move on there needs to be a moratorium on what data needed to be captured, how should they be collected and where should they be stored and analysed. If this is not performed, there will be continuation of discussions that are based on conjecture around the origins of resistance and how to manage this. There is a need to move the conjecture into hypotheses followed by scientific investigation and the sharing of results.

## Footnotes

1 Livestock Unit = 500 kg liveweight.

2 Livestock unit conversion rates: camel = 1.2; cattle and buffalo = 1; equines = 0.7; pigs and other camelids = 0.3; sheep and goats = 0.2; turkeys and dogs = 0.05; chickens, ducks, other poultry, cats = 0.01.

3 The rules that society uses that can include legislation, private standards and cultural norms.

4 http://www.nature.com/news/2011/110216/full/news.2011.46.html

## References

[b1] Acar JF, Moulin G, Page SW, Pastoret PP (2012). Antimicrobial resistance in animal and public health: introduction and classification of antimicrobial agents. Rev. Sci. Tech.

[b2] Castanon JIR (2007). History of the use of antibiotic as growth promoters in European poultry feeds. Poult. Sci.

[b3] Chee-Sanford JC, Aminov RI, Krapac IJ, Garrigues-Jeanjean N, Mackie RI (2001). Occurrence and diversity of tetracycline resistance genes in lagoons and groundwater underlying two swine production facilities. Appl. Environ. Microbiol.

[b4] Codex Alimentarius (2011). Guidelines for Risk Analysis of Foodborne Antimicrobial Resistance (CAC/GL 77–2011).

[b5] Cogliani C, Goossens H, Greko C (2011). Restricting Antimicrobial Use in Food Animals: Lessons from Europe. Banning nonessential antibiotic uses in food animals is intended to reduce pools of resistance genes. Microbe.

[b6] Collignon P, Wegener HC, Braam P, Butler CD (2005). The routine use of antibiotics to promote animal growth does little to benefit protein undernutrition in the developing world. Clin. Infect. Dis.

[b7] Costa E, Re Uwiera R, Kastelic JP, Selinger LB, Inglis GD (2011). Non-therapeutic administration of a model antimicrobial growth promoter modulates intestinal immune responses. Gut. Pathog.

[b8] Danzeisen JL, Bum Kim H, Isaacson RE, Jin Tu Z, Johnson TJ (2011). Modulations of the chicken cecal microbiome and metagenome in response to anticoccidial and growth promoter treatment. PLoS ONE.

[b9] Davies S (2013). The drugs don't work. A global threat.

[b10] Davis B, Winters P, Carletto G, Covarrubias K, Quiñones EJ, Zezza A, Stamoulis K, Azzarri C, DiGiuseppe S (2010). A Cross-Country Comparison of Rural Income Generating Activities. World Dev.

[b11] D'Costa VM, King CE, Kalan L, Morar M, Sung WWL, Schwarz C, Froese D, Zazula G, Calmels F, Debruyne R, Golding GB, Poinar HN, Wright GD (2011). Antibiotic resistance is ancient. Nature.

[b12] Dibner JJ, Richards JD (2005). Antibiotic growth promoters in agriculture: history and mode of action. Poult. Sci.

[b13] Dierikx CM, van der Goot JA, Smith HE, Kant A, Mevius DJ (2013). Presence of ESBL/AmpC-producing *Escherichia coli* in the broiler production pyramid: a descriptive study. PLoS ONE.

[b14] Egervärn M, Börjesson S, Byfors S, Finn M, Kaipe C, Englund S, Lindblad M (2014). *Escherichia coli* with extended-spectrum beta-lactamases or transferable AmpC beta-lactamases and Salmonella on meat imported into Sweden. Int. J. Food Microbiol.

[b15] FAO (2014). http://faostat.fao.org.

[b16] Gerland P, Raftery AE, Sevcikova H, Li N, Gu D, Spoorenberg T, Alkema L, Fosdick BK, Chunn J, Lalic N, Bay G, Buettner T, Heilig GK, Wilmoth J (2014). World population stabilization unlikely this century. Science.

[b17] Gilbert W, Rushton J (2014). Estimating farm-level private expenditure on veterinary medical inputs in England. Vet. Rec.

[b18] Graham JP, Boland JJ, Silbergeld E (2007). Growth promoting antibiotics in food animal production: an economic analysis. Public Health Rep.

[b19] Hennessy DA (2013).

[b20] Herrero M, Thornton PK, Gerber P, Reid RS (2009). Livestock, livelihoods and the environment: understanding the trade-offs. Curr. Opin. Environ. Sustain.

[b21] Huijbers PMC, Graat EAM, Haenen APJ, van Santen MG, van Essen-Zandbergen A, Mevius DJ, van Duijkeren E, van Hoek AHAM (2014). Extended-spectrum and AmpC β-lactamase-producing Escherichia coli in broilers and people living and/or working on broiler farms: prevalence, risk factors and molecular characteristics. J. Antimicrob. Chemother.

[b22] Johnson JR, Sannes MR, Croy C, Johnston B, Clabots C, Kuskowski MA, Bender J, Smith KE, Winokur PL, Belongia EA (2007). Antimicrobial drug-resistant Escherichia coli from humans and poultry products, Minnesota and Wisconsin, 2002-2004. Emerg. Infect. Dis.

[b23] Kaier K, Frank U (2010). Measuring the externality of antibacterial use from promoting antimicrobial resistance. Pharmacoeconomics.

[b24] Kaier K, Moog S (2012). Economic consequences of the demography of MRSA patients and the impact of broad-spectrum antimicrobials. Appl. Health Econ. Health Policy.

[b25] Laube H, Friese A, von Salviati C, Guerra B, Käsbohrer A, Kreienbrock L, Roesler U (2013). Longitudinal monitoring of extended-spectrum-beta-lactamase/AmpC-producing *Escherichia coli* at German broiler chicken fattening farms. Appl. Environ. Microbiol.

[b26] Liu HN, Liu Y, L. Hu L, L. Suo Y, Zhang L, Jin F, Feng XA, Teng N, Li Y (2014). Effects of dietary supplementation of quercetin on performance, egg quality, cecal microflora populations, and antioxidant status in laying hens. Poult. Sci.

[b27] Maron DF, Smith TJS, Nachman KE (2013). Restrictions on antimicrobial use in food animal production: an international regulatory and economic survey. Global. Health.

[b28] Marshall BM, Levy SB (2011). Food animals and antimicrobials: impacts on human health. Clin. Microbiol. Rev.

[b29] Mathews KH (2001). http://www.ers.usda.gov/media/480677/aib766_1_.pdf.

[b30] McEwen SA (2012). Quantitative human health risk assessments of antimicrobial use in animals and selection of resistance: a review of publicly available reports. Rev. Sci. Tech.

[b31] Mclnerney J (1996). Old economics for new problems -livestock disease: presidential address. J. Agric. Econ.

[b32] Niewold TA (2007). The nonantibiotic anti-inflammatory effect of antimicrobial growth promoters, the real mode of action? A hypothesis. Poult. Sci.

[b33] Norwood FB, Lusk JL (2011). Compassion, by the pound: The Economics of Farm Animal Welfare.

[b34] Ostrom E (2010). Beyond Markets and States: Polycentric Governance of Complex Economic Systems. Am. Econ. Rev.

[b35] Pagel SW, Gautier P (2012). Use of anti-microbial agents in livestock. Rev. Sci. Tech.

[b36] Rushton J, Rushton J (2009). Economic Analysis Tools. The Economics of Animal Health and Production.

[b37] Rushton J, Thornton PK, Otte MJ (1999). Methods of economic impact assessment. Rev. Sci. Tech.

[b38] Smith R, Coast J (2013). The true cost of antimicrobial resistance. BMJ.

[b39] Smith RD, Yago M, Millar M, Coast J (2006). A macroeconomic approach to evaluating policies to contain antimicrobial resistance: a case study of methicillin-resistant Staphylococcus aureus (MRSA). Appl. Health Econ. Health Policy.

[b40] Snary EL (2004). Antimicrobial resistance: a microbial risk assessment perspective. The Journal of antimicrobial chemotherapy.

[b41] SOU (1997). http://www.government.se/sb/d/574/a/54899.

[b42] Steinfeld H, Gerber P, Wassenaar T, Castel V, Rosales M, De Haan C (2006). Livestock's long shadow: Environmental issues and options.

[b43] Tansarli GS, Karageorgopoulos DE, Kapaskelis A, Falagas ME (2013). Impact of antimicrobial multidrug resistance on inpatient care cost: an evaluation of the evidence. Expert Rev. Anti. Infect. Ther.

[b44] Thomke S, Elwinger K (1993). Growth promotants in feeding pigs and poultry. I. Growth and feed efficiency responses to antibiotic growth promotants. Annales de zootechnie.

[b45] Tisdell C, Rushton J (2009). Economics of Controlling Livestock Diseases: Basic Theory. Economics of Animal Health & Production.

[b46] Vågsholm I, Höjgård S (2010). Antimicrobial sensitivity–A natural resource to be protected by a Pigouvian tax?. Prev. Vet. Med.

[b47] Warren RE, Ensor VM, O'Neill P, Butler V, Taylor J, Nye K, Harvey M, Livermore DM, Woodford N, Hawkey PM (2008). Imported chicken meat as a potential source of quinolone-resistant Escherichia coli producing extended-spectrum beta-lactamases in the UK. The Journal of antimicrobial chemotherapy.

[b48] Wegener HC (2012). Antibiotic resistance linking human and animal health. Improving Food Safety Through a One Health Approach: Workshop Summary.

[b49] Wolf CA (2013).

